# Bullous Sweet’s Syndrome: Report of an Atypical Case Presenting with Ring-Like, Figurate Lesions

**DOI:** 10.4274/tjh.2015.0202

**Published:** 2017-03-01

**Authors:** Andaç Salman, Aida Berenjian, Ali Eser, Fatma Dilek Kaymakçı, Leyla Cinel, Işık Kaygusuz Atagündüz, Deniz Yücelten, Tülin Ergun

**Affiliations:** 1 Marmara University Faculty of Medicine, Department of Dermatology, İstanbul, Turkey; 2 Marmara University Faculty of Medicine, Department of Hematology, İstanbul, Turkey; 3 Marmara University Faculty of Medicine, Department of Pathology, İstanbul, Turkey

**Keywords:** Bullous, Figurate erythema, myelodysplastic syndrome, Sweet’s syndrome

A 68-year-old woman presented with a 2-month history of erythematous, blistering lesions refractory to systemic antibiotic treatment. Her medical history was insignificant except for long-standing diabetes mellitus, hepatitis C infection, and recently diagnosed myelodysplastic syndrome, refractory anemia with excess blasts-1 (MDS-RAEB-1). She denied any recent intake of drugs prior to the onset of skin lesions. Dermatological examination revealed widespread, erythematous, concentric, circinate large plaques with peripheral bullae formation over the trunk and extremities (Figures 1A and 1B). Laboratory tests disclosed leukocytosis (32x10^9^/L) with neutrophilia (7.2x10^9^/L), anemia (hemoglobin: 76 g/L), thrombocytopenia (16x10^9^/L), elevated levels of C-reactive protein (1133.36 nmol/L) and erythrocyte sedimentation rate (111 mm/h), normal levels of aspartate aminotransferase (0.17 µkat/L) and alanine aminotransferase (0.22 µkat/L), and hepatitis C virus-ribonucleic acid (HCV-RNA) negativity. A punch biopsy was obtained with a differential diagnosis of bullous Sweet’s syndrome (SS) and erythema gyratum repens. Histopathology showed diffuse, dermal inflammatory infiltrate rich in neutrophils with subepidermal blister formation (Figure 2). Clinical and laboratory findings confirmed the diagnosis of bullous SS associated with MDS-RAEB-1. In addition to topical corticosteroids and oral colchicine, treatment with azacitidine led to rapid resolution of the lesions. There was no recurrence of SS until the patient’s death before the second azacitidine cycle.

SS is characterized by erythematous, tender plaques and papules involving the head, neck, and upper extremities [1,2]. It may be associated with infections, hematologic malignancies, inflammatory bowel disease, and drugs [2]. SS may also be associated with chronic active hepatitis; however, normal liver function tests, HCV-RNA negativity, and the temporal relationship between skin lesions and hematological findings in our case make this unlikely. Although pseudovesicular appearance due to severe edema can be seen in SS, bullae formation with figurate and ring-like lesions is rare [3,4,5]. Figurate lesions without bullae in SS were previously reported in a patient with no associated disease [3]. In conclusion, the diagnosis of SS should be kept in mind in patients with erythema gyratum repens-like or concentric blistering lesions.

## Figures and Tables

**Figure 1:**
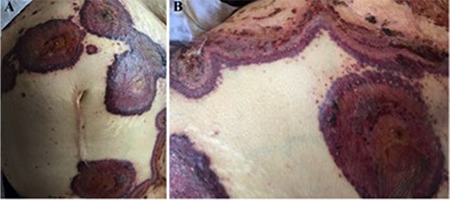
Widespread, erythematous, ring-like plaques with peripheral blisters on the trunk (A and B).

**Figure 2:**
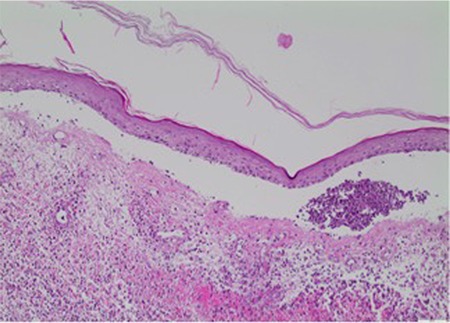
Dermal infiltrate rich in neutrophils with subepidermal blister formation (H&E, 20^x^).
